# Positive correlational shift between crevicular antimicrobial peptide LL-37, pain and periodontal status following non-surgical periodontal therapy. A pilot study

**DOI:** 10.1186/s12903-023-03023-w

**Published:** 2023-05-28

**Authors:** David Madruga, Miguel M Garcia, Luca Martino, Haidar Hassan, Ghada Elayat, Lucy Ghali, Laura Ceballos

**Affiliations:** 1 Area of Stomatology, Department of Nursing and Stomatology, Faculty of Health Sciences, Universidad Rey Juan Carlos (URJC), Avda. de Atenas s/n, Alcorcón, E-28922 Spain; 2 Area of Pharmacology, Nutrition and Bromatology, Department of Basic Health Sciences, Faculty of Health Sciences, Universidad Rey Juan Carlos (URJC), Unidad Asociada I+D+i Instituto de Química Médica (IQM) CSIC-URJC, Avda. de Atenas s/n, Alcorcón, E-28922 Spain; 3 High Performance Experimental Pharmacology Research Group, Universidad Rey Juan Carlos (PHARMAKOM), Alcorcón, Spain; 4 Grupo Multidisciplinar de Investigación y Tratamiento del Dolor (i+DOL), Universidad Rey Juan Carlos (URJC), Alcorcón, Spain; 5 Area of Signal Theory and Communications, Department of Signal Theory and Communications and Telematics Systems and Computing, Higher Technical School of Telecommunications Engineering, Universidad Rey Juan Carlos (URJC), Cam. del Molino, 5, Fuenlabrada, E-28942 Spain; 6 High Performance Data Science and Signal Processing for Networks and Society research group, Universidad Rey Juan Carlos (DSSP), Fuenlabrada, Spain; 7 Academic Plastic Surgery, School of Medicine and Dentistry, Blizard Institute, Barts and The London, Queen Mary University of London, London, E1 2AD UK; 8 Department of Natural Sciences, Faculty of Science and Technology, Middlesex University, London, NW4 4BT UK; 9 Department of Pathology, Faculty of Medicine, Tanta University, El Bahr St, Tanta, 31111 Egypt; 10 High Performance Development and Innovation in Dental Biomaterials Research Group, Universidad Rey Juan Carlos (IDIBO), Alcorcón, Spain

**Keywords:** Periodontitis, Gingival crevicular fluid, Cathelicidin LL-37, Interleukins, Pain measurement

## Abstract

**Background:**

Periodontitis has a high prevalence and uncertain recurrence. Unlike the pro-inflammatory cytokine profile, little is known about the anti-inflammatory cytokine and antimicrobial peptide overview following treatment. The present study aimed to evaluate if any of the antimicrobial peptide LL-37, interleukin (IL) 4, 10 and 6 together with the volume of gingival crevicular fluid (GCF) and total protein concentration in GCF could be used as correlative biomarkers for the severity in periodontitis as well as prognostic factors in the management of the disease.

**Methods:**

Forty-five participants were recruited and allocated to the healthy (15), Stage I-II (15) or Stage III-IV periodontitis (15) group. Along with periodontal examination, GCF samples were obtained at baseline and 4–6 weeks following scaling and root planing (SRP) for the periodontitis groups. GCF samples were analyzed by ELISA kits to quantify LL-37 and IL-4, -6 and − 10. One-way ANOVA followed by Dunnett’s test was used to determine differences among the three groups at baseline. Two-way ANOVA followed by Sidak’s post-hoc test was used to compare between pre- and post-SRP in the two periodontitis groups.

**Results:**

The amount of GCF volume was significantly correlated to the severity of periodontitis and decreased following SRP, particularly in the Stage III-IV group (*p* < 0.01). The levels of LL-37, IL-6, and pain and periodontal clinical parameters were significantly correlated to the severity of periodontitis. IL-4 and IL-10 in the periodontitis groups were significantly lower than the healthy group (*p* < 0.0001) and barely improved following SRP up to the level of the healthy group.

**Conclusions:**

With the limitations of this study, crevicular LL-37 may be a candidate for a biomarker of periodontitis and the associated pain upon probing.

**Trial registration:**

The study was registered in clinical trials.gov, with number NCT04404335, dated 27/05/2020.

**Supplementary Information:**

The online version contains supplementary material available at 10.1186/s12903-023-03023-w.

## Background

Periodontal disease is a multifactorial condition of inflammatory origin that results in a dysbiosis of the oral biofilm microbiome [1]. There are mainly two types of periodontal disease: gingivitis and periodontitis. Unlike the former, periodontitis involves loss of attachment, subsequent alveolar bone and may lead to tooth loss [2].

Under healthy conditions, the highly vascularized periodontal tissue constitutively delivers nutrients, serum proteins and other macromolecules responsible for immune surveillance and avoidance of plaque biofilm overgrowth into the gingival sulcus [3]. However, perturbations on this well-controlled niche environment may trigger an increased directional migration of neutrophils into the gingival pocket and activation of resident lymphocytes and macrophages. In turn, this results in the exudate of a gingival crevicular fluid (GCF) containing a plethora of inflammatory mediators including antimicrobial peptides and cytokines [4]. Neutrophils and macrophages are major components of the innate system, playing a crucial role in inflammation responses to both infection and tissue injury [5, 6]. Some of the mediators secreted by these cells may trigger both an inflammatory and a nociceptive response [7].

Among these antimicrobial peptides, cathelicidin LL-37 has drawn special attention over the past few years as a pivotal player in conditions that course with inflammatory-evoked tissue degeneration, particularly in the digestive and respiratory systems [8, 9]. Previous evidence suggests that neutrophils accumulate a precursor inactive form (hCAP-18) within secretory granules and, upon cell activation during the inflammatory process, LL-37 is released following enzymatic cleavage [10]. In this regard, LL-37 would exert chemoattractant and immunomodulating activities toward other immune cells [11], although interpretations on its meaning remain uncertain. Some authors point at deficiency in salivary LL-37 as a likely reason for periodontitis [12, 13], whereas others have suggested that LL-37 levels in GCF increase in the presence of gingival inflammation [14]. In addition to these roles, a recent study has suggested that pain could be independent from inflammation in a model of LL-37-induced interstitial cystitis and painful bladder syndrome in mouse [15]. Hence it is worth relevant to further investigate LL-37 as a potential pain-triggering molecule.

Along similar lines, recent development of highly sensitive biochemical tests has enabled tuned comparisons of inflammatory mediator levels in whole saliva and GCF samples between healthy individuals and patients with periodontitis. However, most studies still focus on pro-inflammatory cytokines (e.g., IFN-γ, IL-1β, IL-2, IL-6, IL-8, IL-12, IL-17, TNF) [16–19], generally outnumbered in healthy subjects. It is worth mentioning that, although mostly regarded as a pro-inflammatory cytokine, IL-6 also has many regenerative or anti-inflammatory activities [20]. On the contrary, there are fewer data on anti-inflammatory cytokines (e.g., IL-4, IL-9, IL-10, IL-13) [21], and besides, conclusions stay controversial for some of them [1, 18]. For example, IL-4 and IL-10 are mainly produced by lymphocytes, more precisely, by Th2 cells, major components of the immune adaptive system. Some authors have described absence or very low levels of IL-4 in both healthy individuals and periodontitis patients [18], whereas others report lower levels in gingival samples of periodontitis subjects [22]. In a similar way, some authors suggest higher salivary IL-10 levels in periodontitis than in healthy individuals [1], whereas others have described just the opposite [23]. The reason for these discrepancies is still unclear, but the type of sample (GCF or saliva), the genetic background and other factors could be implicated.

Gaining a clear understanding of the mechanisms that underlie a pathological condition can help decide the optimal therapeutic strategy. In this respect, mechanical debridement by scaling and root planing (SRP) is considered the gold standard treatment for most patients with periodontitis [21]. Although it is an effective method, pain and dental fear have been reported to be associated with the procedure [24, 25]. In addition, it may be necessary to implement other adjuvant therapies (e.g., systemic and local antibiotics, laser-supported therapy, antimicrobial photodynamics) to increase the effectiveness of the treatment in patients refractory to SRP [26, 27]. The reason for this variable efficacy is uncertain but treated periodontal pockets have been suggested to be rapidly re-colonized by periodontal pathogens from yet-untreated periodontal pockets and oral niches [28, 29]. This implies that, in some patients, conventional non-surgical periodontal therapy may not be efficient for long and the gingival inflammation may reoccur.

The aim of the present study was to investigate any shifts in personalized antimicrobial peptide (LL-37) and cytokine (IL-4, IL-6 and IL-10) profiles in GCF in periodontitis patients subsequent to SRP.

## Materials and methods

### Experimental design

The study was designed as a prospective cohort, three-arm parallel-group study with a roughly one-and-a-half-month follow-up period. All participants were examined by one examiner (DM). Based on the clinical and radiographic evaluation, the participants were distributed into the healthy, Stage I-II, and Stage III-IV periodontitis groups. Along with periodontal examination, GCF sampling, self-reported pain measurements, and a detailed medical history were taken. The two periodontitis groups received full-mouth SRP. Oral hygiene instructions were given for all dental patients. Periodontitis patients were additionally asked to rinse twice daily with 0.12% chlorhexidine for the following seven post-SRP days. Re-examination and new sample collection were carried out after 4–6 weeks from the two periodontitis groups. Total protein quantification in GCF and ELISA assays were performed by another examiner (MMG). Triple blinding was ensured to avoid any bias in the study. To do so, all images and samples were labeled with an alphanumeric identifier. Numbers corresponded to individual participants allocated in order of registration, whereas two different letters were assigned based on pre-SRP or post-SRP therapy. The medical and dental histories of each patient were only assigned a number to be referred by.

### Participants

A total of 45 individuals (21 males and 24 females; with age range 23–77 years old; mean age 46.02 (SD 16.14)) were recruited from the University Clinic at the Faculty of Health Sciences of Universidad Rey Juan Carlos between June 2021 and June 2022 following a screening evaluation including full-mouth periodontal probing and radiographic examination. The inclusion criteria were aged 18 or over, with no previous history of periodontal treatment in the last six months and no medical or dental history that could contraindicate periodontal treatment. Exclusion criteria included current pregnancy or lactation, immunological disorders, periodontal treatment within the last 6 months, oral contraception and antibiotic usage within the last 3 months, and infections (such as HIV, hepatitis, and tuberculosis). Patients with clinical sings of gingivitis (with a probing depth (PD) = 1-3 mm but no signs of clinical attachment loss/bone loss), were excluded from the study.

**Fig. 1:**
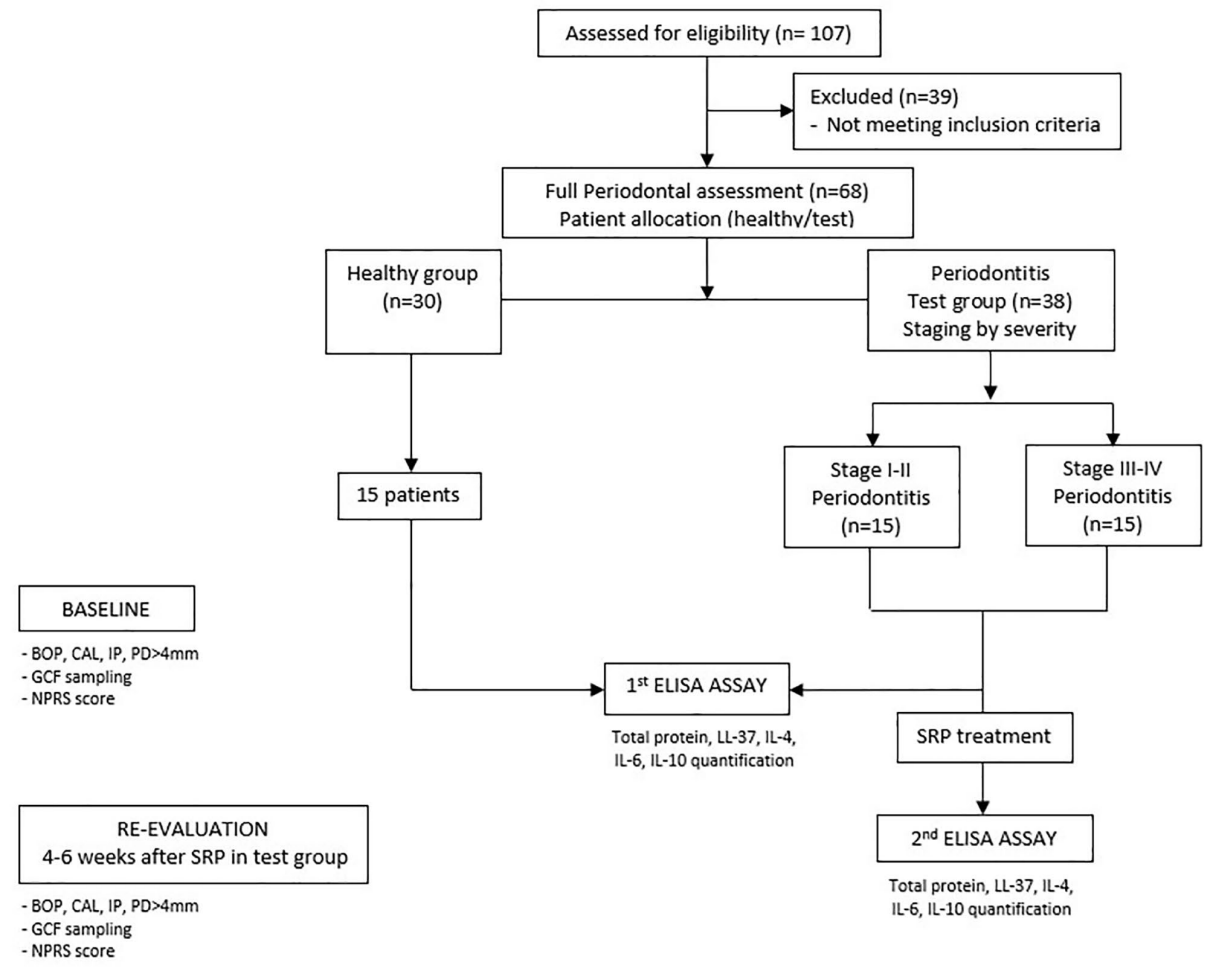
**Flow diagram of the selection criteria.** n = number of patients

A flow-diagram of the selection criteria is shown in Fig. 1. All study procedures were performed in compliance with a protocol approved by the Ethical Committee of Universidad Rey Juan Carlos (protocol number 2711201916719) and followed STROBE guidelines for reporting observational studies.

Periodontitis patients were defined as patients presenting gingival inflammation with clinical attachment loss/bone loss, according to the 2018 Classification of Periodontal and Peri-implant Diseases and Conditions [30]. After concluding a diagnosis for periodontitis, a stage was assigned based on the degree of clinical attachment loss (CAL) and radiographic bone loss (RBL) (1-2 mm or 3-4 mm CAL for Stage I or II and ≥ 5 mm for Stage III-IV). Healthy subjects were defined as: no sites with interdental attachment loss, PD = 1-3 mm and BOP < 10% [31].

Plaque index (PI) was also recorded at baseline in both healthy and periodontitis I-II – III-IV groups and after SRP therapy in periodontitis groups. The index was calculated by obtaining 6-point measurements (3 buccal and 3 palatal/lingual) of each tooth present. The record obtained was then divided by the number of available sites examined and multipled by 100 to express it as an index [32].

### Evaluation of pain

Pain was evaluated using an 11-point self-reported numeric pain rating scale (NPRS) [33]. A 0–10 score, in which 0 represented “no pain” and 10 indicated “the most pain imaginable”, was recorded from the participants, who verbally selected the value that was most in line with the intensity of oral pain that they had experienced during periodontal probing. NPRS was implemented on first attendance (pre-SRP) and on the re-examination day.

### Gingival crevicular fluid sampling and processing

Gingival crevicular fluid was sampled and processed as previously described [34] with minor modifications. Briefly, supragingival plaque was carefully removed before GCF sampling. Collection sites were then isolated using cotton rolls and dried with air jets, and corresponded to those showing deepest pocket depths with bleeding on probing (BOP) with clinical signs of inflammation along with intraoral periapical radiographic confirmation of bone loss. In this regard, all teeth surfaces (buccal, mesio-buccal and disto-buccal) were more or less similarly represented within and between the different study groups (Supplementary Table 1). PerioPaper® GCF collection strips (Oraflow Inc; Hewlett, NY, USA; 593520) were overlaid and placed at the gingival crevice region until mild resistance was felt. Then, left in place for 30 s to prevent any mechanical irritation. Any strip contaminated with blood was excluded. The volume was calculated with the help of a Periotron® 8010 (Oraflow Inc; Hewlett, NY, USA; 593480) device and corresponded to an inflammatory exudate that was used as an index for the degree of periodontal inflammation. The Periotron® device was calibrated according to the supplier’s indications. In short, a standard calibration curve was obtained by using a 4th order polynomial to plot the relationship between Periotron scores and fluid volume. Measurements of known volumes at various intervals (0, 0.25, 0.50, 0.75, 1.00 and 1.25 µl) were taken on PerioPaper® GCF collection strips in triplicate and the average Periotron Score at each interval was recorded. Data points were plotted on a graph and a linear equation was used to best represent the data. Interpolating from the resulting standard curve was then performed to convert Periotron Scores to sample volumes. Periotron zero was periodically checked and readjusted when necessary. Additionally, to remove any residual crevicular fluid or debris, sensors were gently wiped with a paper tissue wetted with phosphate buffer saline (1x PBS, pH 7.2) before any new measurement [16]. The same representative periodontal pocket from patients with periodontitis stage I-II or III-IV was compared pre-SRP and post-SRP and to samples obtained from healthy individuals.

Immediately after sample collection, the strips were stored in sterile test tubes (Eppendorf Corporate; Hamburg, Germany; 0030125150) with an added volume of 500 µl of 1x phosphate buffered-saline (PBS, pH 7.2) and refrigerated at 4 ºC until the following day. A vortex-type stirrer was used to thoroughly mix the exudate with the dilutant for 2 min and the sample was then centrifuged at 10,000 rpm for 10 min. Subsequently, the paper strips were removed and the remaining volumes preserved at -80 ºC until their use.

Additionally, total protein concentration for each sample was measured in duplicate or triplicate as required using the NanoDrop™ 2000 Spectrophotometer (Thermo Fisher Scientific, Wilmington, DE, USA) following the supplier’s instructions. Fresh prepared 1x PBS (pH 7.2) was used as sample blank.

### Quantification of cathelicidin LL-37 and cytokines IL-4, IL-6, IL-10

Commercially available human antibacterial peptide LL-37 enzyme-linked immunosorbent assay (ELISA) kit (Cusabio Technology LLC; Houston, TX, USA; CSB-E14948h) and IL-4, IL-6 and IL-10 high sensitivity ELISA kits (Diaclone SAS; Besançon, France; 850.890, 950.035 and 850.880, respectively) were used to determine the concentration of each compound. Assays were carried out in accordance with the manufacturers’ instructions and all measurements were done in duplicate. The results were expressed as total amount (pg) of cathelecidin LL-37 or cytokine per site. Samples with protein levels below the assay’s detection limit were scored as 0.

### Statistical analyses

The hypothesis referred to whether we could find differences among healthy, Stage I-II, and Stage III-IV periodontitis. In this regard, changes in PDs and BOP (before vs. after SRP therapy) were considered the primary outcome variables. For an effect size between 0.41 and 0.57 based on previous works [35, 36], α = 0.05, power = 0.8, and 2 measurements (before and after treatment), a total sample size between 27 and 48 individuals was calculated (G*Power 3.1.9.4, Heinrich-Heine-Universität Düsseldorf, Germany). A sample size of 45 subjects was selected and the same number of participants per group were enrolled.

Descriptive parameters consisted of demographical data, oral health habits, and risk factors associated with periodontal disease (age, diabetes, smoking, periodontal measurements) were presented as numbers or means and standard deviations (SD). The normality of all data was assessed by the Shapiro-Wilk test. One-way ANOVA followed by Dunnett’s multiple comparisons test was used to determine statistical significance between healthy individuals and each periodontitis group at baseline. Two-way ANOVA (treatment x group) followed by Sidak’s multiple comparisons test were used to determine statistical significance between pre- and post-SRP effects in the Stage I-II and III-IV groups. To study the possible correlations between each molecular mediator and pain, two-tailed Spearman’s correlation analyses were used. All analyses were performed using statistical software (GraphPad Prism 8.0.1, San Diego, CA, USA). A level of significance of P < 0.05 was assumed.

## Results

### Description of the patients’ sample

Table 1 summarizes the characteristics of the study population. Each group consisted of 15 subjects with a total of 45 participants. The mean ages of the Stage I-II (54.27 (14.42)) and Stage III-IV (51.93 (11.13)) periodontitis groups were significantly higher (*p* < 0.0001 and *p* < 0.001, respectively) compared to the healthy group (31.87 (12.72)).

**Table 1:** Characteristics of the sample according to common risk factors associated to periodontitis

	Healthy			Stage I-II			Stage III-IV		
	women	men	sum	women	men	sum	women	men	sum
age (y) (mean (SD))	29.29 (7.41)	34.13 (16.25)	31.87 (12.72)	56.63 (10.99)	51.57 (18.12)	54.27 (14.42)^++++^	52.33 (9.59)	51.33 (14.11)	51.93 (11.13)^+++^
Diabetes (n)	0	0	0	0	0	0	0	1	1
Smoker (n)	0	1	1	2	0	2	0	3	3

Data are expressed as mean (SD). ^+^*p* < 0.05, ^+++^*p* < 0.001, ^++++^*p* < 0.0001 vs. healthy individuals. One-way ANOVA followed by Dunnett’s multiple comparisons test

### Periodontal status and clinical effects of periodontal therapy

Periodontal clinical parameters for the Stage I-II and Stage III-IV periodontitis group are presented in Table 2. Percentage of sites with BOP and PI significantly decreased after periodontal treatment in both Stage I-II (BOP: 43.00 (29.77) vs. 19.67 (18.03), *p* < 0.001; PI: 33.67 (23.75) vs. 16.73 (12.00), *p* < 0.01) and III-IV periodontitis patients (BOP: 62.07 (29.14) vs. 37.67 (24.03), *p* < 0.0001; PI: 47.93 (29.78) vs. 27.80 (18.74), *p* < 0.01). However, differences between Stage I-II and Stage III-IV periodontitis were not found to be statistically significant.

**Table 2:** Periodontal clinical parameters (mean (SD)) of the sites sampled for molecular analyses in the two clinical groups, at baseline and 4–6 weeks after therapy

	Stage I-II		Stage III-IV	
	Pre-SRP	Post-SRP	Pre-SRP	Post-SRP
**Clinical parameters**				
PD (mm)	4.53 (0.52)	3.40 (0.83)***	6.47 (1.51)	5.27 (1.39)***
CAL (mm)	2.13 (0.64)	1.42 (0.51)*	5.94 (1.06)	3.78 ± (0.94)****
**% of sites with**				
PD > 4 mm	12.40 (12.99)	7.00 (10.09)*	47.93 (23.72)	39.13 (27.58)**
BOP	43.00 (29.77)	19.67 (18.03)***	62.07 (29.14)	37.67 (24.03)****
PI	33.67 (23.75)	16.73 (12.00)**	47.93 (29.78)	27.80 (18.74)**

PD = probing depth; CAL = clinical attachment loss; BOP = bleeding on probing; PI = plaque index. * *p* < 0.05, ** *p* < 0.01, *** *p* < 0.001, **** *p* < 0.0001 pre-SRP vs. post-SRP. Two-way ANOVA followed by Sidak’s multiple comparisons test

### Evaluation of pain

**Fig. 2:**
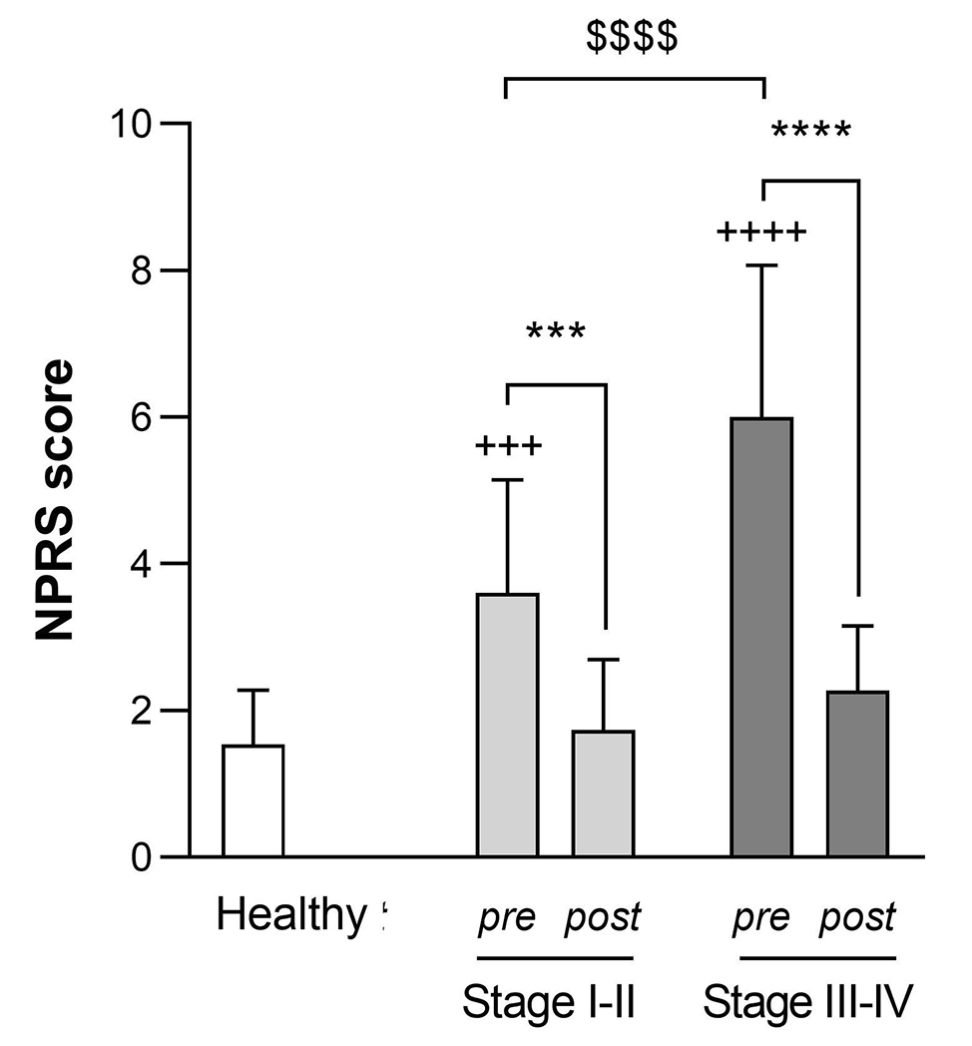
NPRS score for each group before and after SRP therapy. ^**^p < 0.01, ^***^p < 0.001, ^****^p < 0.0001 pre-SRP vs. post-SRP; ^$$$$^*p* < 0.0001 vs. Periodontitis Stage I-II; two-way ANOVA followed by Sidak’s multiple comparisons test. ^+++^*p* < 0.001, ^++++^*p* < 0.0001 vs. healthy group; one way ANOVA followed by Dunnett’s multiple comparisons test. Data are expressed as mean (SD). pre = pre-SRP, post = post-SRP, NPRS = numeric pain rating scale

The mean NPRS scores were 1.53 (0.74), 3.60 (1.55) and 6.00 (2.07) for healthy, Stage I-II and Stage III-IV periodontitis groups, respectively, at baseline. Statistically significant differences were observed when compared to the healthy group (Stage I-II, *p* < 0.001; Stage III-IV, *p* < 0.0001) and also between two periodontitis groups (*p* < 0.0001). These values decreased significantly after SRP treatment in both periodontitis groups (Stage I-II, *p* < 0.001; Stage III-IV, *p* < 0.0001). Furthermore, there were no statistically significant differences post-SRP as compared to healthy individuals for either stage (I-II: 1.73 (0.96) and III-IV: 2.27 (0.88)) (Fig. 2).

### GCF volume and total protein concentration

**Fig. 3:**
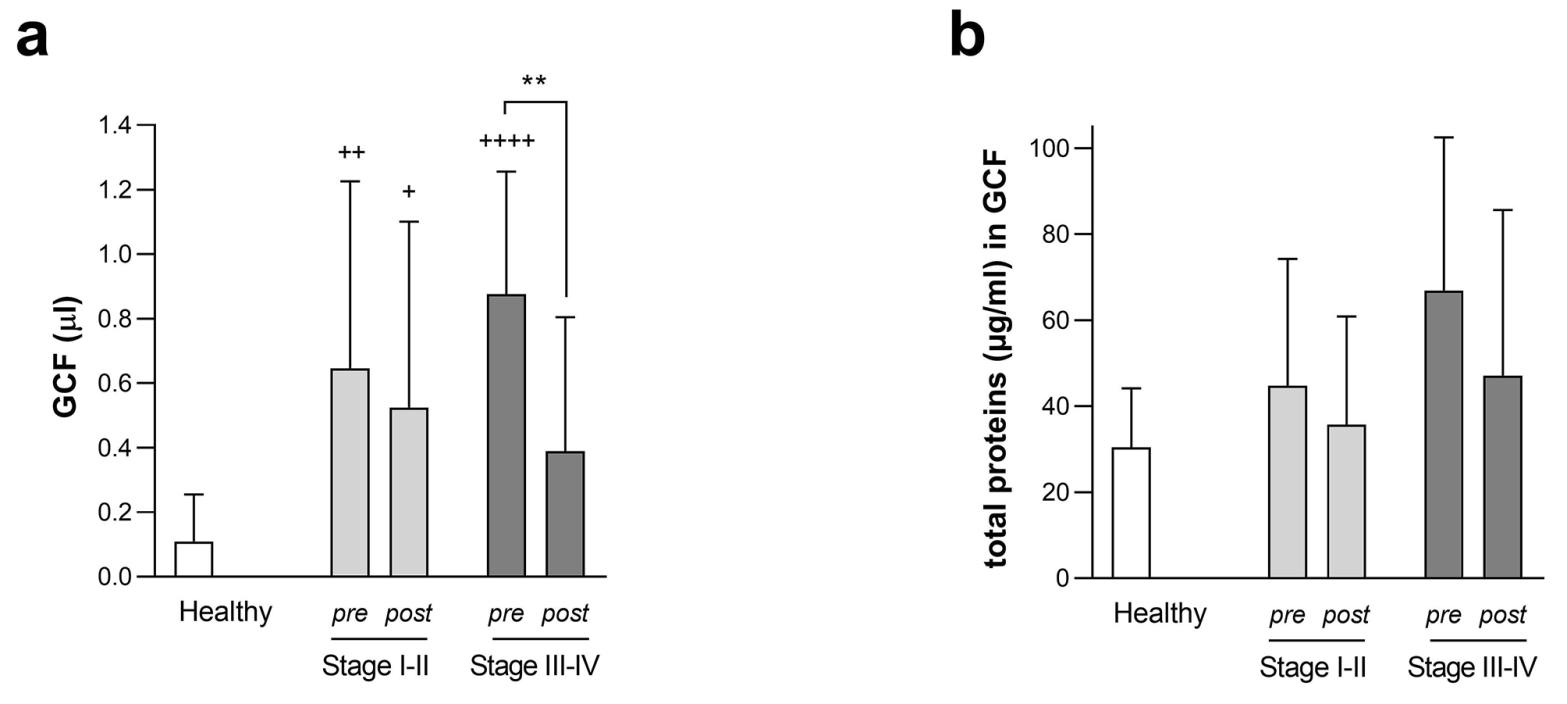
**GCF volume (µl) (a) or total protein concentration in GCF (µg/ml) (b) for each group before and after SRP therapy.** Data are expressed as mean (SD). * *p* < 0.05, ** *p* < 0.01 pre-SRP vs. post-SRP; two-way ANOVA followed by Sidak’s multiple comparisons test. ^+^*p* < 0.05, ^++^*p* < 0.01, ^++++^*p* < 0.0001 vs. healthy group; one-way ANOVA followed by Dunnett’s multiple comparisons test. pre = pre-SRP, post = post-SRP, GCF = gingival crevicular fluid

GCF volume was significant lower in the healthy group (0.11 (0.15)) than in the Stage I-II (0.65 (0.58), *p* < 0.01) and III-IV (0.88 (0.38), *p* < 0.0001) periodontitis groups, with no significant differences between the two periodontitis groups. These volumes decreased following SRP treatment (I-II: 0.52 (0.58), ns; III-IV: 0.39 (0.42), *p* < 0.01). Particularly, the Stage III-IV periodontitis group showed no significant differences when compared to the healthy group (*p* < 0.05) after SRP (Fig. 3a).

At baseline, total protein concentration in GCF (µg/ml) was higher for periodontitis groups, 44.79 (29.48) (Stage I-II) and 66.93 (35.54) (Stage III-IV), than for the healthy group, 30.41 (13.74). However, no statistically significant differences were found between any of the stages and the healthy group. Similarly, although not statistically significant, 4–6 weeks after SRP treatment, total protein concentration in GCF was reduced as compared to baseline in both groups (Stage I-II: 35.70 (25.16), Stage III-IV: 47.16 (38.46)) (Fig. 3b).

### Levels of antimicrobial peptide LL-37, pro/anti-inflammatory cytokine IL-6 and anti-inflammatory cytokines IL-4 and IL-10 in GCF

**Fig. 4:**
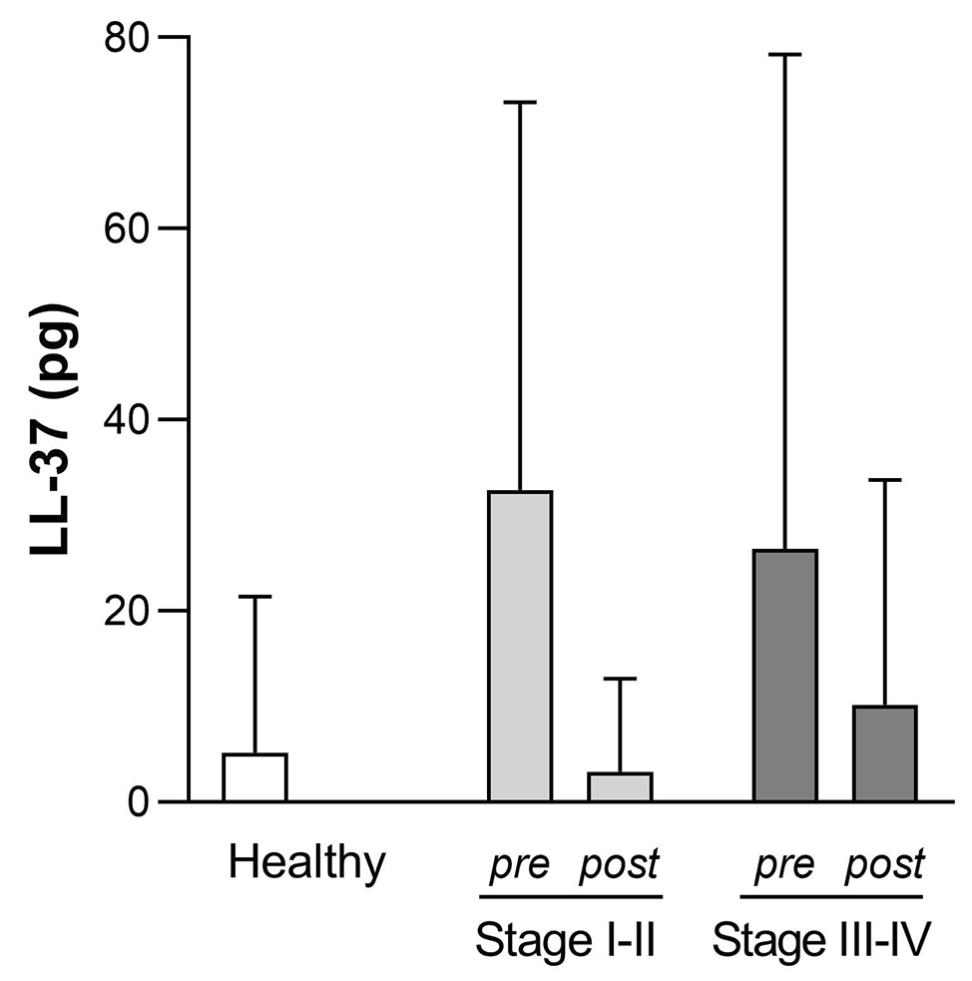
**Cathelicidin LL-37 levels in GCF (pg/site) for each group before and after SRP therapy.** Data are expressed as mean (SD).

LL-37 levels in GCF (pg/site) were 9.96 (25.28) pg, 32.62 (40.61) pg and 26.47 (51.79) pg for the healthy, Stage I-II and III-IV periodontitis groups, respectively. After SRP, values for Stage I-II and III-IV periodontitis were 3.20 (9.71) pg and 17.16 (35.43) pg, respectively. Although no statistically significant differences were found as compared to the healthy group nor between pre- and post-SRP therapy, LL-37 levels were elevated in both periodontitis groups and reduced after SRP (Fig. 4).

**Fig. 5:**
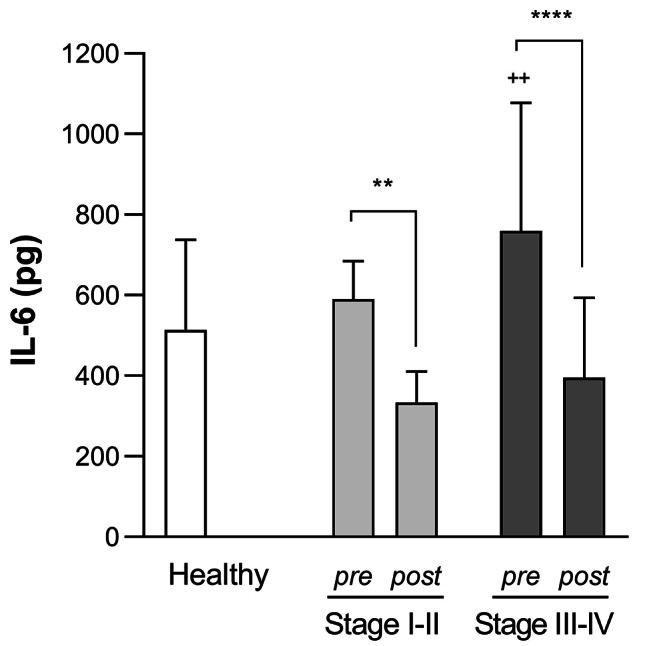
**IL-6 levels in GCF (pg/site) for each group before and after SRP therapy.** Data are expressed as mean (SD). ** *p* < 0.01, **** *p* < 0.0001 pre-SRP vs. post-SRP. Two-way ANOVA followed by Sidak’s multiple comparisons test. ^++^*p* < 0.01 vs. healthy group. One-way ANOVA followed by Dunnett’s multiple comparisons test

IL-6 levels in GCF (pg/site) were elevated for both Stage I-II (590.30 (93.86) pg) and III-IV (759.20 ± (318.00) pg) groups as compared to the healthy group (513.90 (223.70) pg) at baseline, although statistically significant differences were only found for Stage III-IV periodontitis (*p* < 0.01). Following SRP, IL-6 levels in GCF significantly decreased (Stage I-II: 333.50 (76.68) pg, *p* < 0.01; Stage III-IV: 395.10 (198.60) pg, *p* < 0.0001) (Fig. 5).

**Fig. 6:**
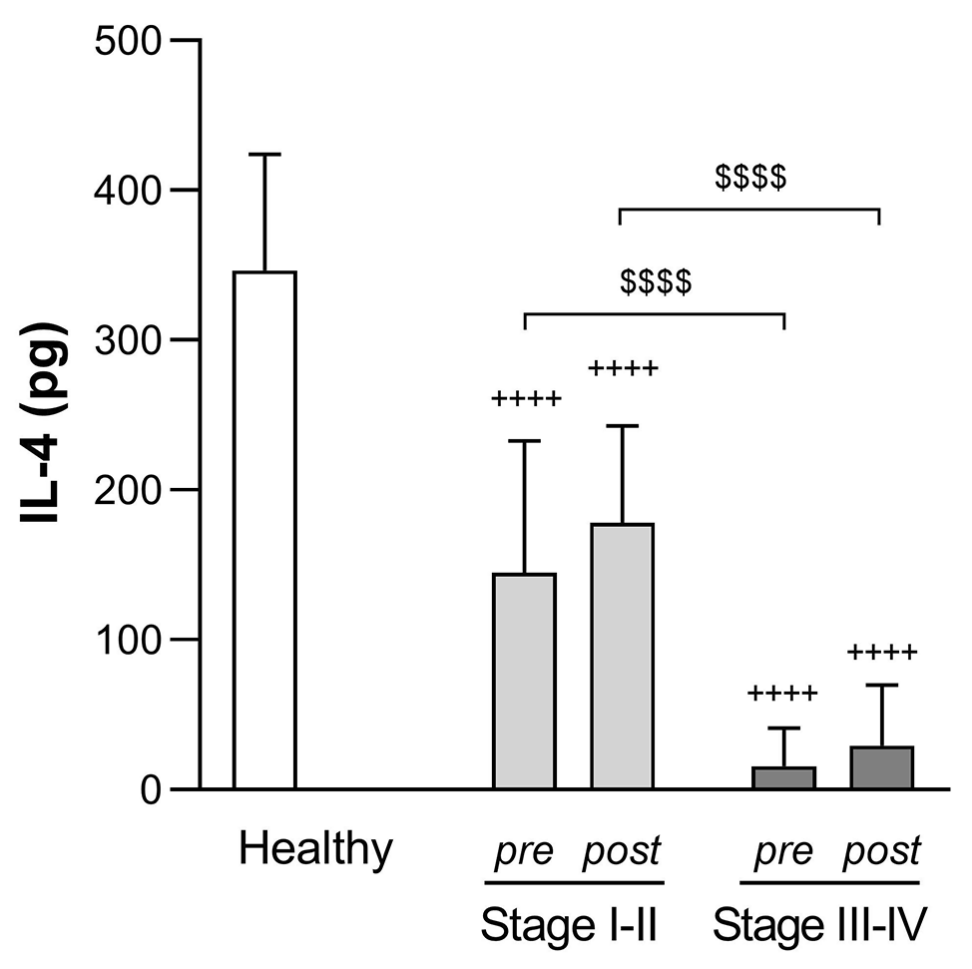
**IL-4 levels in GCF (pg/site) for each group before and after SRP therapy.** Data are expressed as mean (SD). ^$$$$^*p* < 0.0001 vs. Stage I-II periodontitis. Two-way ANOVA followed by Sidak’s multiple comparisons test. ^++++^*p* < 0.0001 vs. healthy group. One-way ANOVA followed by Dunnett’s multiple comparisons test

Unlike antimicrobial peptide LL-37 and IL-6, IL-4 and IL-10 levels in GCF were substantially lower in the periodontitis groups as compared to healthy individuals at baseline. IL-4 levels in GCF (pg/site) were 346.30 (77.81) pg, 144.90 (87.84) pg and 15.80 (25.19) pg for the healthy, Stage I-II and Stage III-IV periodontitis groups, respectively. Statistically significant differences existed between healthy and patient groups (*p* < 0.0001) and between patient groups (*p* < 0.0001). These values remained unvaried after SRP treatment in both patient groups (Stage I-II: 178.30 (64.30) pg; Stage III-IV: 29.35 (40.64) pg) (Fig. 6).

**Fig. 7:**
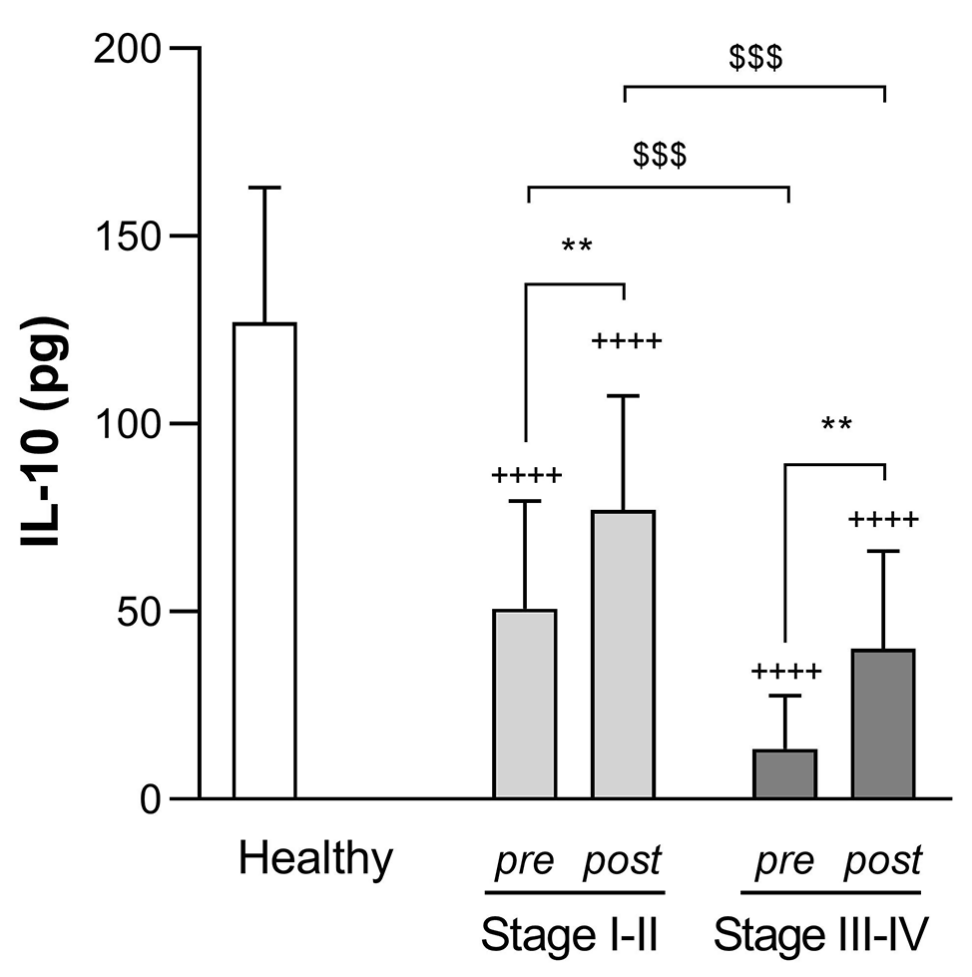
**IL-10 levels in GCF (pg/site) for each group before and after SRP therapy.** Data are expressed as mean (SD). ** *p* < 0.01 pre-SRP vs. post-SRP; ^$$$^*p* < 0.001 vs. Stage I-II periodontitis. Two-way ANOVA followed by Sidak’s multiple comparisons test. ^++++^*p* < 0.0001 vs. healthy group. One-way ANOVA followed by Dunnett’s multiple comparisons test

IL-10 levels in GCF (pg/site) were 127.20 (35.76) pg, 50.76 (28.74) pg and 13.49 (14.04) pg for healthy, Stage I-II and Stage III-IV periodontitis groups, respectively. Evident statistically significant differences existed between healthy and patient groups (*p* < 0.0001) and between patient groups themselves (*p* < 0.001). These values were significantly increased after SRP treatment in both Stage I-II (77.11 (30.27) pg, *p* < 0.01) and Stage III-IV periodontitis groups (40.06 (25.95) pg, *p* < 0.01). However, differences as compared to the healthy group (*p* < 0.0001) or between both patient groups (*p* < 0.001) remained unchanged (Fig. 7).

As a secondary objective, the association between pain and the aforementioned molecular mediators was further addressed. Distribution of all molecular mediators (pg) departed significantly from normality. Therefore, two-tailed Spearman’s correlation analyses were used to analyze the associations between NPRS scores and the aforementioned parameters. Correlations were found for all two set of variables (Table 3). As pain scores increased, so did LL-37 (pg) and IL-6 (pg) levels in GCF (r = 0.3125 and 0.4284, respectively). On the contrary, IL-4 (pg) and IL-10 (pg) levels decreased accordingly (r = -0.4743 and − 0.5194, respectively).

**Table 3:** Spearman’s rank correlation between NPRS score and the molecular mediators LL-37, IL-6, IL-4 and IL-10

Non-parametric Spearman’s correlation			
	95% CI	r(df)	*p*
LL-37 (pg)	0.0767–0.5153	r(68) = 0.3125	< 0.01 (**)
IL-6 (pg)	0.2047–0.6096	r(66) = 0.4284	< 0.001 (***)
IL-4 (pg)	-0.6402 – -0.2661	r(70) = -0.4743	< 0.001 (***)
IL-10 (pg)	-0.6733 – -0.3224	r(71) = -0.5194	< 0.001 (***)

r, correlation coefficient; 95% CI, 95% confidence interval

## Discussion

Periodontitis represents a public health concern due to its high prevalence and uncertain recurrence after conventional non-surgical treatment. In addition, therapy outcomes may be variable; however, the subjacent cause is yet to be unveiled [37–39]. While non-surgical therapy is effective to manage periodontitis, follow-up studies are scarce, which may account for the unsolved problem of the condition regarding its chronicity and recurrence despite the initial treatment efficacy.

Pain levels upon periodontal probing can be influenced by gingival inflammation; pro-inflammatory cytokines are involved in the process of pathological pain [40, 41] and in this respect, antimicrobial peptide LL-37 has been recently suggested to have a functional role in pain signalling [15]. On the other hand, anti-inflammatory cytokines like IL-4 or IL-10 are thought to directly limit the bioavailability of specific pro-inflammatory cytokines, hence plausibly improving pain management [42].

In this study, NPRS scores were significantly higher for the Stage III-IV group than for the healthy group. After 4–6 weeks following conventional non-surgical treatment, no differences were found in the NPRS scores among all groups. SRP therapy might reduce gingival inflammation so that the pain levels reduced to the level of the healthy group.

The quantification of GCF volume and the characterization of the mediators in the GCF are common methods to assess the severity of the periodontal disease [10, 14, 42–45]. Higher GCF volumes corresponded to more severe periodontitis and the GCF volume decreased following SRP treatment. A similar trend was observed for total protein quantification in the GCF, although no statically significant differences were observed. Crevicular total protein levels may be used as indicators of the disease severity in absence of the Periotron device, but a larger sample size might be required to obtain statistically significance between the healthy and periodontitis groups.

Of all proteins that are present in the GCF, we focused on a series of “anti”-mediators. We found increased levels of LL-37 (pg/site) in the presence of gingival inflammation. In the two periodontitis groups, 4–6 weeks post-SRP LL-37 levels were comparable to the healthy group. The similar trend as explained for LL-37 was observed for the pro-/anti-inflammatory cytokine IL-6. However, we are uncertain to whether IL-6 was playing a pro-, anti-inflammatory role, or both here. Moreover, although IL-6 always responded to consistent detectable baseline levels, LL-37 was practically absent in healthy and periodontitis patients after SRP therapy, appearing only in the untreated periodontitis patients. On the other hand, anti-inflammatory cytokines IL-4 and IL-10 showed a negative correlation with periodontitis. This was in contradiction to that reported by Archana et al. [23], but in line with that shown by Pradeep et al. [46] and Varma et al. [1]. Similarly, although Al-Hamoudi [21] reported increased levels of IL-4 and IL-10 compared with their respective baseline values three months following SRP therapy, our study showed that SRP therapy had little or no effect on the levels of IL-4 at baseline. This controversy may be explained by the different follow-up time that we used, since the trends in both studies are in fact comparable.

The different outcomes encountered herein for LL-37 and IL-6 and IL-4 and IL-10 may explain the intricate mechanisms behind the disease and its management. As recently suggested by Tokajuk et al. [47], LL-37 may postulate as an optimal candidate for tracking the evolution of the disease at short/middle-term, since unlike anti-inflammatory IL-4 and IL-10, LL-37 levels decreased to healthy conditions (almost zero) following SRP.

In order to further analyze the clinical outcome, we evaluated the association between pain and the aforementioned molecular mediators in the GCF. Pain scores increased when IL-6 levels (*p* < 0.001) and LL-37 levels (*p* < 0.01) increased. This would indicate that LL-37 level in GCF could also be taken as a reliable direct pain biomarker in periodontal disease. This is in line with that reported by Jia et al. [15] in a model of LL-37-induced interstitial cystitis and painful bladder syndrome in mouse. Although a previous study reported the relationship between levels of pain intensity and pro-inflammatory cytokines in acute severe pericoronitis [48], to our knowledge this is the first study indicating a positive correlational shift between periodontal pain and crevicular LL-37 following non-surgical conventional treatment. On the other hand, although the correlation between pain and IL-4 or IL-10 levels remained negative (*p* < 0.001), contrary to the distributional shift observed in pain scores, IL-4 and IL-10 were barely affected following SRP therapy.

The biggest weakness of the study is the short follow-up period, which have not allowed to account for significant changes in the prognosis of the disease after SRP in periodontitis patients. The collection of GCF samples may have been affected by prosthesis or orthodontic devices in some patients. The age distribution was not even; our recruitment resulted in an older mean age for the periodontitis groups than for the healthy group, which makes it uncertain to state the exact cause of the uneven molecular levels in GCF for the periodontitis groups. Larger samples would be required to conduct robust statistics.

## Conclusions

The current study highlights potential positive correlations between clinical indicators of periodontal status, pain upon probing, and the levels of antimicrobial peptide LL-37 and IL-6 in GCF. Longitudinal prospective cohort studies with a large sample size are suggested to investigate LL-37 as a reliable molecular biomarker for the therapeutic efficacy and the prognosis of periodontitis.

## Electronic supplementary material

Below is the link to the electronic supplementary material.

**Supplementary Table 1.** Representation of the different teeth surfaces in each study group.

## Data Availability

All data and materials used are available by the corresponding author on reasonable request.

## List of Abbreviations

BL: Bone loss
BOP: Bleeding on probing
CAL: Clinical attachment level
CEJ: Cement-enamel junction
GCF: Gingival crevicular fluid
NPRS: Numeric pain rating scale
PBS: Phosphate buffer saline
PD: Probing depth
PI: Plaque index
SRP: Scale and root planning
