# Supplementary material for: Positive correlational shift between crevicular antimicrobial peptide LL-37, pain and periodontal status following non-surgical periodontal therapy. A pilot study
Source: BMC Oral Health. 2023 May 28;23:335. doi: 10.1186/s12903-023-03023-w (PMC10226254; doi:10.1186/s12903-023-03023-w)
Supplement: Supplementary file 1 — Supplementary Table 1. Representation of the different teeth surfaces in each study group. [file 12903_2023_3023_MOESM1_ESM.docx]

**Supplementary Table 1. Representation of the different teeth surfaces in each study group.** Data are expressed as number of patients. A Chi-Square Goodness of Fit Test was performed to determine whether the proportion of the different teeth surfaces was equal between healthy and each periodontitis group. The proportions did not differ by tooth surface, X^2^(8, 45) = 5.544, P = 0.698, so the null hypothesis of no relationship at the given significance level can be accepted. Overall, all teeth surfaces were similarly represented between the different study groups. Additional Chi-Square Tests of Independence were performed to assess the relationship between each two Stages. Given that Chi-Square tests can be unstable when there are small numbers of observations, we combined Stage I-II and III-IV, resulting in no significant tooth surface differences between both, X^2^(4, 45) = 4.094, P = 0.393. Again, all teeth surfaces were similarly represented within the periodontitis group. B = buccal ; MB = mesio-buccal; DB = disto-buccal.

|  | **Healthy** |  | **Stage I-II** | **Stage III-IV** | **Stage I** | **Stage II** | **Stage III** | **Stage IV** |
| --- | --- | --- | --- | --- | --- | --- | --- | --- |
| **B** | 4 | | 3 | 7 | 2 | 1 | 4 | 3 |
| **MB** | 8 | | 6 | 5 | 2 | 4 | 2 | 3 |
| **DB** | 3 | | 6 | 3 | 3 | 3 | 1 | 2 |
| **Total** | 15 | | 15 | 15 | 7 | 8 | 7 | 8 |
